# Serotonergic Dysfunction in Amyotrophic Lateral Sclerosis and Parkinson's Disease: Similar Mechanisms, Dissimilar Outcomes

**DOI:** 10.3389/fnins.2018.00185

**Published:** 2018-03-20

**Authors:** Yannick Vermeiren, Jana Janssens, Debby Van Dam, Peter P. De Deyn

**Affiliations:** ^1^Laboratory of Neurochemistry and Behavior, Department of Biomedical Sciences, Institute Born-Bunge, University of Antwerp, Antwerp, Belgium; ^2^Department of Neurology and Alzheimer Research Center, University of Groningen and University Medical Center Groningen, Groningen, Netherlands; ^3^Department of Neurology, Memory Clinic of Hospital Network Antwerp (ZNA) Middelheim and Hoge Beuken, Antwerp, Belgium

**Keywords:** serotonin (5-HT), dopamine, glutamate, amyotrophic lateral sclerosis (ALS), Parkinson's disease (PD), raphe nuclei, basal ganglia

## Abstract

Amyotrophic lateral sclerosis (ALS) and Parkinson's disease (PD) share similar pathophysiological mechanisms. From a neurochemical point of view, the serotonin (5-hydroxytryptamine; 5-HT) dysfunction in both movement disorders—related to probable lesioning of the raphe nuclei—is profound, and, therefore, may be partially responsible for motor as well as non-motor disturbances. More specifically, in ALS, it has been hypothesized that serotonergic denervation leads to loss of its inhibitory control on glutamate release, resulting into glutamate-induced neurotoxicity in lower and/or upper motor neurons, combined with a detrimental decrease of its facilitatory effects on glutamatergic motor neuron excitation. Both events then may eventually give rise to the well-known clinical motor phenotype. Similarly, disruption of the organized serotonergic control on complex mesencephalic dopaminergic connections between basal ganglia (BG) nuclei and across the BG-cortico-thalamic circuits, has shown to be closely involved in the onset of parkinsonian symptoms. Levodopa (L-DOPA) therapy in PD largely seems to confirm the influential role of 5-HT, since serotonergic rather than dopaminergic projections release L-DOPA-derived dopamine, particularly in extrastriatal regions, emphasizing the strongly interwoven interactions between both monoamine systems. Apart from its orchestrating function, the 5-HT system also exerts neuroprotective and anti-inflammatory effects. In line with this observation, emerging therapies have recently focused on boosting the serotonergic system in ALS and PD, which may provide novel rationale for treating these devastating conditions both on the disease-modifying, as well as symptomatic level.

## Background

The neurotransmitter serotonin (5-hydroxytryptamine; 5-HT) is produced in the raphe nuclei (RN), a moderately sized cluster of caudal and rostral neurons (B1-B9) found in the brainstem (Dahlstroem and Fuxe, 1964). Axons arising from the caudal group (B1-B4) form a descending system projecting to the spinal cord, cerebellum, pontine, and midbrain structures, whereas ascending fibers emanating from the more rostral clusters (B5-B9) connect with the cerebral cortex, (hypo)thalamus, basal ganglia and hippocampus among others. About roughly 300,000 5-HT-containing neurons in the human brain bear a tremendous number of collateral branches so that the serotonergic system densely innervates nearly all brain regions (Jacobs and Azmitia, 1992). It is, therefore, not surprising that this extensive neuronal network is implicated in the regulation of numerous physiological events, such as hormone secretion, sleep-wake cycle, motor control, immune system functioning, nociception, food intake, energy balance/metabolism, cardiovascular/respiratory functioning, body temperature, affect/aggression, consciousness, learning, and memory (Ciranna, 2006; Sandyk, 2006). Serotonin receptors are subdivided into seven families (5-HT_1−7_), based on structural, biochemical and pharmacological characteristics, resulting into 14 subtypes (5-HT_1A/1B/1D/1e/1F_, 5-HT_2A/2B/2C_, 5-HT_3_, 5-HT_4_, 5-HT_5A/5b_, 5-HT_6_, and 5-HT_7_). With the sole exception of 5-HT_3_, which belongs to the ligand gated ion channels, all 5-HT receptors are G protein-coupled receptors, mediating a variety of physiological and behavioral functions (Filip and Bader, 2009). Regarding amyotrophic lateral sclerosis (ALS) and Parkinson's disease (PD) pathophysiology, especially 5-HT_1A/1B_ and 5-HT_2A/2B/2C_ seem crucial (Cummings et al., 2013; Miyazaki et al., 2013; De Deurwaerdère and Di Giovanni, 2016; El Oussini et al., 2016). In short, the 5-HT_1A_ receptor is expressed in the RN as a presynaptic autoreceptor, while it also functions as a postsynaptic heteroreceptor in areas of the limbic system, such as the prefrontal cortex, hippocampus, lateral septum, and amygdala, as well as in (hypo)thalamus, and basal ganglia (Hoyer et al., 1994). Activation of 5-HT_1A_ autoreceptors on the cell bodies or dendrites of the RN neurons exerts inhibitory feedback in response to local 5-HT release. The 5-HT_1B_ receptors are centered on axonal terminals of (non)serotonergic neurons, mainly found in the basal ganglia and substantia nigra (SN) (Bonaventure et al., 1997). Interestingly, it has been indicated that 5-HT_1B_ receptors are preferentially located on presynaptic terminals of ɤ-amino-butyric acid (GABA)ergic neurons, and, it has also been suggested that thalamostriatal and/or corticostriatal glutamatergic neurons express presynaptic 5-HT_1B_ receptors (Bonaventure et al., 1998). In contrast, the 5-HT_2A_ receptor is found mainly in the periphery and neocortical areas, where they are implicated in the pathogenesis of schizophrenia and hallucinations (Burnet et al., 1995; Hannon and Hoyer, 2008). These receptors are highly expressed in both pyramidal cells and GABAergic interneurons. Moreover, cerebral 5-HT_2B_ receptors are present in the cerebellum, cerebral cortex, hypothalamus, corpus callosum and amygdala, causing anxiolytic effects among others (Duxon et al., 1997). Noteworthy, this receptor subtype is likely to be expressed by RN neurons, where this autoreceptor might play a role in the regulation of the serotonin transporter (SERT) (Diaz et al., 2012). Next, the 5-HT_2C_ subtype is widely distributed throughout the brain and has been proposed as the main mediators of the different actions of 5-HT in the central nervous system (Hannon and Hoyer, 2008). Additionally, 5-HT_2C_ receptors are commonly found in the choroid plexus, where they modulate cerebrospinal fluid (CSF) production (Pasqualetti et al., 1999).

The serotonergic system is organized in such a way that it exerts widespread effects on targeted neurons, such as motor neuron excitability threshold control, and interacts with many other neurotransmitters, including dopamine (DA), noradrenaline (NA), glutamate, GABA, and various peptides (Ciranna, 2006). Remarkably, 5-HT also plays an important part in the development of the embryonic nervous system, which relates to neurite outgrowth and other aspects of neuronal differentiation, including synaptogenesis (Lauder, 1990). Given its complex but critical modulating characteristics, 5-HT can be regarded as one of the principal orchestrators of the central nervous system, with a very significant role in motor activity. In PD and ALS, two invariably fatal neurodegenerative conditions, the motor and non-motor features have been partially attributed to disease-related malfunctioning of this overseeing neurotransmitter system.

## Serotonergic degeneration in PD and ALS

Staging of brain pathology in PD demonstrated an early involvement of Lewy body depositions within the RN. In more detail, Halliday et al. (1990) firstly described a 56% loss of serotonergic neurons in the median RN of PD compared to control brain. Afterwards, Braak et al. (2003) determined six stages in the evolution of PD-related pathology, with lesions being present in the median RN in the caudal brainstem already from stage two onwards. Furthermore, 5-HT depletion was observed in various target areas of the RN, such as in the basal ganglia, hypothalamus, hippocampus, and prefrontal cortex (Fahn et al., 1971; Shannak et al., 1994). This was later confirmed by *in vivo* imaging studies, revealing new insights. For instance, Politis et al. (2010) applied ^11^C-DASB-PET to early-stage PD patients, and demonstrated reduced SERT binding in the caudate nucleus, (hypo)thalamus, and anterior cingulate cortex, whereas PD subjects with established disease showed additional ^11^C-DASB binding reductions in the putamen, insula, posterior cingulate cortex, and, prefrontal cortex. Further binding reductions were noticed in the ventral striatum, RN, and amygdala of advanced PD patients. Interestingly, the loss of SERT binding in the RN occurred in later stages, pointing to an earlier loss of serotonergic projections instead of the neurons themselves.

**Figure 1:**
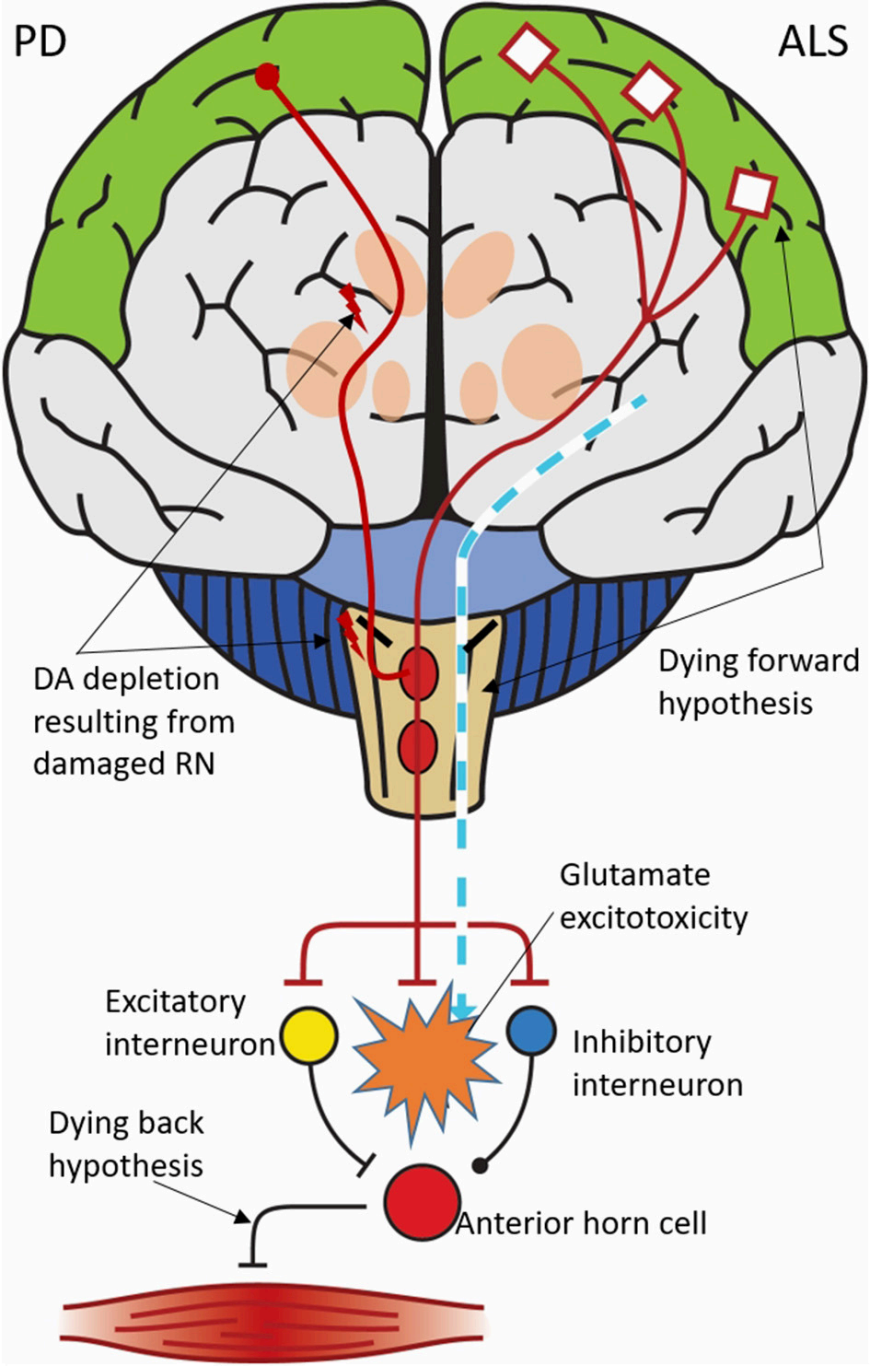
Schematic representation of dysfunctional serotonergic pathway interactions in ALS and PD, mediated by lesioned raphe nuclei (RN) centered in the brainstem. In summary, serotonergic loss in amyotrophic lateral sclerosis (ALS) brain and subsequent loss of its inhibitory control on glutamate release cause glutamate-induced excitotoxicity leading to upper/lower motor neuron damage. In this regard, the *dying forward* hypothesis proposes that ALS is a disorder primarily of the corticomotoneurons, with anterior horn cell degeneration propagated via an anterograde glutamate-dependent excitotoxic process. In contrast, the *dying back* hypothesis proposes that ALS begins within the muscle or neuromuscular junction, with pathogens being retrogradely transported from the neuromuscular junction to the cell body where they may exert their deleterious effects. Simultaneously, this figure illustrates the pathophysiological serotonergic-dopaminergic interactions on the striatal level in Parkinson's disease (PD), where lesioning of the RN (red spheres) in addition to dopamine (DA) depletion in the striatum and substantia nigra (black-bolded dashes) give rise to a net decreased activity of the motor cortex. Adapted from Vucic et al. (2013), ©2013 with permission from BMJ Publishing Group Ltd.

In ALS, distribution patterns of TAR DNA-binding protein (TDP)-43 intraneuronal inclusions have only recently been investigated, summing up into a total of four discriminative neuropathological stages (Brettschneider et al., 2013). Notably, it has been theorized that sites with projections to the cortex remain intact in ALS, unlike those receiving corticofugal axonal projections, supporting the hypothesis of prion-like propagation of TDP-43, potentially from the motor cortex downwards (dying forward/back hypotheses, Figure 1). In agreement, the upper RN with diffuse cortical projections barely become affected by TDP-43 pathology in ALS, which is in great contrast with PD or Alzheimer's disease (AD) (Braak et al., 2013). Nevertheless, a marked reduction in both cortical and RN 5-HT_1A_ receptor binding (21%) has been observed (Turner et al., 2005), and, several studies previously evidenced decreased levels of 5-HT, 5-hydroxyindoleacetic acid (5-HIAA; main metabolite of 5-HT) or tryptophan (precursor of 5-HT) in CSF, plasma, and/or spinal cord (Monaco et al., 1979; Ohsugi et al., 1987; Bertel et al., 1991; Sofic et al., 1991). Platelet 5-HT levels also positively correlated with survival in ALS subjects (Dupuis et al., 2010). Consequently, it has been postulated that 5-HT_1A/2_ receptor (anta)agonists, 5-HT precursors [e.g., 5-hydroxytryptophan (5-HTP)] (Turner et al., 2003) or 5-HT_2B/C_ receptor inverse agonists (Dentel et al., 2013) might improve locomotor function and even strategically interfere with ALS disease course. On the whole, the serotonergic theory in ALS has gained renewed interest especially due to several recent publications (Dentel et al., 2013; El Oussini et al., 2016, 2017).

## 5-HT and the control of motor neuron excitability: possible implications

The indolamine 5-HT has facilitatory effects on glutamatergic motor neuron excitation by augmenting weak or polysynaptic inputs, bringing motor neurons to threshold. This effect on spinal motor neurons is exerted through 5-HT_1/2_ receptors (for review: Sandyk, 2006). In ALS, serotonergic denervation has been hypothesized to lead to significant loss of inhibitory control on glutamate release, via decreased binding on presynaptic 5-HT_1B_ receptors, triggering glutamate-induced neurotoxicity, and, eventually, rapid-onset loss of upper and lower motor neurons (Muramatsu et al., 1998). Upper motor neurons are glutamatergic neurons located in layer V of the motor cortex, project to spinal motor neurons through the corticospinal tract, and are the major source of descending motor commands for voluntary movement (Lemon, 2008). Meanwhile, progressive degeneration of 5-HT neurons in the motor cortex, RN and their projections may lead to a compensatory increase in glutamate excitation (Bertel et al., 1991), adding up to the clinical motor phenotype (Figure 1). Conversely, motor neuron groups such as the oculomotor, trochlear, and abducens nuclei, and the cerebellum, which only receive sparse serotonergic innervation, appear more resistant to the process of neurodegeneration in ALS. Moreover, it is possible that differences between bulbar and spinal ALS in the course of the disease may be related to the degree of cerebral 5-HT depletion, which seems more extensive in the bulbar subtype (Turner et al., 2005). Additionally, 5-HT is a precursor of melatonin, which inhibits glutamate release and glutamate-induced neurotoxicity (Zhang et al., 1999).

One of the possible implications of serotonergic degeneration with regard to motor symptoms in ALS, is spasticity (Dentel et al., 2013; El Oussini et al., 2017). For instance, El Oussini et al. (2017) recently demonstrated that degeneration of brainstem 5-HT neurons in transgenic SOD1 (G37R) mice, more particularly the dorsal and median RN, induced spasticity. This hyperreflexia is able to compensate for motor deficits, allowing the maintenance of motor function after disease onset. Spasticity is a painful symptom which can severely restrict quality of life on a daily basis. Remarkably, SB206553 administration, a 5-HT_2B/C_ receptor inverse agonist, completely abolished spasticity symptoms (Murray et al., 2010; El Oussini et al., 2017). The authors further stress that selective degeneration of the RN might directly lead to motor neuron hyperexcitability and spasticity—rather than degeneration of upper motor neurons in the cerebral motor cortex.

## Related treatment options

So far, riluzole and edaravone—of which latter drug has recently been FDA-licensed in the US and Japan (Hardiman and van den Bergh, 2017)—only modestly improve motor symptoms and daily functioning in ALS patients, but with a reasonable safety profile of riluzole 100 mg daily (Miller et al., 2007). However, the treatment regimen for edaravone is inconvenient and costly (Hardiman and van den Bergh, 2017). Riluzole acts as an n-methyl-d-aspartate (NMDA)-receptor antagonist, whereas edaravone is a free radical scavenger. Both mechanisms of action thus support the glutamate excitotoxicity-driven hypothesis.

As for clinical trials with serotonergic therapies, Meininger et al. (2004) carried out two randomized, double-blind, placebo-controlled multicenter studies (phase III) with xaliproden, a 5-HT_1A_ receptor agonist which has neurotrophic and neuroprotective effects, to assess its safety and efficacy at two doses. ALS patients were randomly assigned to placebo, 1 or 2 mg xaliproden orally once daily as a monotherapy (867 patients), or, to the same regimen with addition of riluzole 50 mg (1,210 patients). In the end, however, the primary outcome measures (time to death, tracheostomy, or permanent assisted ventilation) did not reach statistical significance. There only was a therapeutic benefit on the second outcome measure, i.e., vital capacity (maximum volume of air exhaled at a steady state after maximum inhalation of a single breath) at the 1 and 2 mg dose without riluzole, which let the authors conclude that xaliproden does not effectively slow down disease progression. In short, strong evidence is currently lacking and insufficient regarding the potential benefits of serotonergic therapies in ALS, despite of its remarkable 5-HT-related pathophysiological characteristics described above.

Finally, monoamine oxidase-B (MAO-B) inhibitors such as deprenyl, rasagiline, or selegiline affect the release and increase the content of not only DA and NA, but also of 5-HT (for review: Finberg, 2014). MAO-B inhibitors also have neuroprotective properties. Following the introduction of rasagiline to the therapeutic armamentarium for PD, various successes have been reported (Rascol et al., 2005, 2011). In ALS, results are less consistent, with selegiline treatment having no significant effect on the rate of clinical progression or outcome in ALS as evidenced by Lange et al. (1998), whereas deprenyl and rasagiline seem more promising, but necessitate further scrutiny (Jossan et al., 1994; Macchi et al., 2015).

## Serotonergic modulation of basal ganglia and mesencephalic dopaminergic activity in PD

The basal ganglia (BG) are composed of the striatum (caudate nucleus and putamen), subthalamic nucleus (STN), internal and external globus pallidus (GPi/e) and SN, and are part of the BG-cortico-thalamic circuits. This highly organized network is important for motor control, emotion, and cognition. It has been firmly established that BG nuclei receive vast serotonergic input mainly coming from the rostral RN clusters (B7), with effects on mesencephalic dopaminergic activity depending on the specific nucleus and its receptor distribution (excitatory via 5-HT_1A/1B/2A/3/4/7_ and inhibitory via 5-HT_2C/6_ receptors (Paolucci et al., 2003; Miguelez et al., 2014; De Deurwaerdère and Di Giovanni, 2016). In PD, lesioning of the RN in addition to DA depletion in the striatum and SN—particularly of the pars compacta (SNc)—are hallmarks of the disease, leading to overactivation of the output regions of the BG, i.e., GPi and SN pars reticulata (SNr), which contain large GABAergic neurons. This cascade results in a net decreased activity of the supplementary motor areas, premotor, and primary motor cortices, triggering parkinsonian symptoms (Albin et al., 1989; Figure 1). Overall, the loss of 5-HT neurons is not as profound as the loss of DA neurons, and may not be sufficient to cause motor or non-motor symptoms *per se*, however, both systems closely interact, and combined depletion certainly seems to aggravate the situation, as was shown in a parkinsonian rat model (Delaville et al., 2012). Moreover, 5-HT and 5-HIAA levels, as well as SERT expression, are reduced in various BG nuclei (Scatton et al., 1983; Guttman et al., 2007; Kish et al., 2008), and the serotonergic system is strongly involved in the mechanism of action of antiparkinsonian therapeutics, such as levodopa (L-DOPA), and high frequency stimulation of the STN (Navailles and De Deurwaerdère, 2012).

## L-DOPA actions via serotonergic nerve terminals in PD: the influential effect of 5-HT

Levodopa (L-DOPA), the metabolic precursor of DA, is a well-established symptomatic treatment for the motor deficits in PD. Paradoxically, L-DOPA-induced dyskinesia (LID), as well as hallucinations, are unfortunate but more or less inevitable corollaries of its long-term administration (De Deurwaerdère et al., 2017). Despite the traditional assumption that L-DOPA is transformed in residual striatal dopaminergic neurons into DA, interestingly, a more important role for serotonergic than dopaminergic projections has been identified for the increase of extracellular DA, predominantly in prefrontal cortex, nucleus accumbens, STN, hippocampus, and additional extrastriatal regions (De Deurwaerdère et al., 2017). Briefly, 5-HT neurons convert exogenous L-DOPA into DA and store neo-synthesized DA into vesicles for exocytosis via vesicular monoamine transporter 2, as was originally shown in rats (Arai et al., 1995). Since the distribution of 5-HT terminals in the brain is very different from dopaminergic innervation, the magnitude of effect in extrastriatal regions is tremendous compared to physiological conditions, especially at low L-DOPA doses, so that 5-HT in fact controls the dopaminergic output in a state and region-dependent manner (Navailles and De Deurwaerdère, 2012).

Latter phenomenon has even led to the assumption that future envisaged pharmacotherapeutic strategies to treat LID should specifically aim at controlling L-DOPA-stimulated DA release from extrastriatal 5-HT neurons (Miguelez et al., 2014; De Deurwaerdère et al., 2017). Recently, the use of 5-HTP (Tronci et al., 2013) or 5-HT_1A/B_ receptor agonists (e.g., eltoprazine or buspirone; Svenningsson et al., 2015; De Deurwaerdère et al., 2017)—influencing DA release indirectly via action on the overall 5-HT tone—has been proposed. As for exacerbation of psychosis by L-DOPA treatment—attributed to excessive DA release in the mesolimbic areas rather than the motor striatum, mediated by hypersensitive 5-HT signaling—a favorable role for 5-HT_2A_ receptor inverse agonists (e.g., pimavanserin) or 5-HT_2A_ antagonists (e.g., low doses of clozapine) has likewise been demonstrated (Cummings et al., 2013). These findings suggest that the serotonergic system may even adapt to the lack of DA by adopting anatomical and functional transformations in PD.

## Alterations in other monoamine neurotransmitter systems

NA levels have been previously reported to be significantly increased in the cervical, thoracic and lumbar spinal cord of ALS patients compared to controls (Bertel et al., 1991), with highest concentrations measured in ventral and intermediate gray matter. In CSF, a similar increase has been noted (Ziegler et al., 1980). Independently of 5-HT, NA increases the excitability of motor neurons to glutamate (White and Neuman, 1980). Bertel et al. (1991) further discussed that in all probability, it is unlikely that the noradrenergic changes were due to the effect of tissue shrinkage—since concentrations were expressed in ng/g wet weighed tissue—but rather a consequence of denser noradrenergic (neosympathetic) innervation, such as from sprouting of noradrenergic fibers into affected areas. In PD, the noradrenergic dysfunction has been investigated in more detail. In summary, α-synuclein depositions in the locus coeruleus (stage 2), the brain's main source of NA, have been evidenced to precede that of the SN (stage 3) (Braak et al., 2003). Consequently, neuronal loss in this noradrenergic nucleus and the accompanying noradrenergic deficiency both on the central and peripheral level have been related to various motor and non-motor (cognitive) symptoms, including the progression to (prodromal) dementia (Vermeiren and De Deyn, 2017).

A potential dopaminergic deficit in ALS has only been scarcely investigated, with significantly reduced striatal DA transporter expression in patients with bulbar- or limb-onset compared to controls ([I-123]-IPT-SPECT) (Borasio et al., 1998), while in drug-naïve, sporadic ALS patients, decreased striatal D2-receptor binding could be partially reversed by riluzole (Vogels et al., 1999). On the contrary, no differences in spinal DA concentrations were found between ALS and control subjects (Bertel et al., 1991). No research has been performed yet with regard to 5-HT-DA interactions in mesencephalic brain areas or BG nuclei, but a study by Xu et al. (2017) observed an abnormal cortical-BG network in ALS after applying resting state fMRI and voxel-wise network analysis.

## The non-motor outcomes of serotonergic dysfunction in ALS and PD

New findings point at an important link between non-linear progressive degeneration of serotonergic terminals and non-motor disturbances in PD, such as depression, fatigue, weight loss, and anxiety (Politis and Niccolini, 2015; Huot et al., 2017). Similarly, cognitive impairment and dementia are major issues in PD, and might be ascribed to serotonergic dysfunction too (Huot et al., 2017). In this respect, a phase 2 trial is currently evaluating the safety, tolerability and efficacy of SYN120, a dual 5-HT_6_/5-HT_2A_ antagonist, in 80 PD dementia patients over a 16-week period [SYNAPSE; NCT02258152 (clinicaltrials.gov)]. As for ALS, fatigue and abnormal peripheral glucose metabolism have been suggested (Reyes et al., 1984). Major depressive disorder, in which an ~12% reduction of cortical 5-HT_1A_ binding is seen in non-ALS cases (Sargent et al., 2000), is relatively rare in ALS patients, even in later stages (Goldstein et al., 2006). More recently, Vercruysse et al. (2016) indicated that serotonergic axonal loss in the arcuate nucleus of the hypothalamus in combination with decreased hypothalamic 5-HT levels primarily caused a melanocortin deficit in mutant SOD1 (G86R) mice, which contributed to dysregulated food intake/weight loss.

Furthermore, self-referential thinking (i.e., reflecting one's mental self) is a key cognitive process which seems to be regulated by 5-HT_1A_ receptors within the default mode network, which comprises the precuneus, posterior cingulate cortex, medial prefrontal cortex, and, the temporoparietal junction (Hahn et al., 2012). In this regard, Fomina et al. (2017) observed electroencephalography correlates (bandpower) of self-referential thinking in the medial prefrontal cortex of healthy individuals, but not ALS patients. The authors concluded that these cognitive abnormalities, such as anosognosia, may well be in compliance with the proposed serotonergic theory in ALS.

## (DIS)similarities: 5-HT as a crucial disease modifier

The process of normal, healthy aging has complex effects on central and peripheral serotonergic transmission. Accumulating (pre)clinical evidence suggests a linear and gradual decline of 5-HT connections from the RN, as well as altered SERT and 5-HT_1A/2A_ receptor expressions in multiple brain regions (Rodríguez et al., 2012). However, in ALS and PD, RN neuronal loss and/or loss of serotonergic projections due to marked and early TDP-43 and α-synuclein depositions in target areas might cause major imbalance in monoaminergic neurotransmission across the brain (Turner et al., 2005; Dentel et al., 2013; Politis and Niccolini, 2015), accounting for numerous motor, behavioral and cognitive dysfunctions.

**Figure 2:**
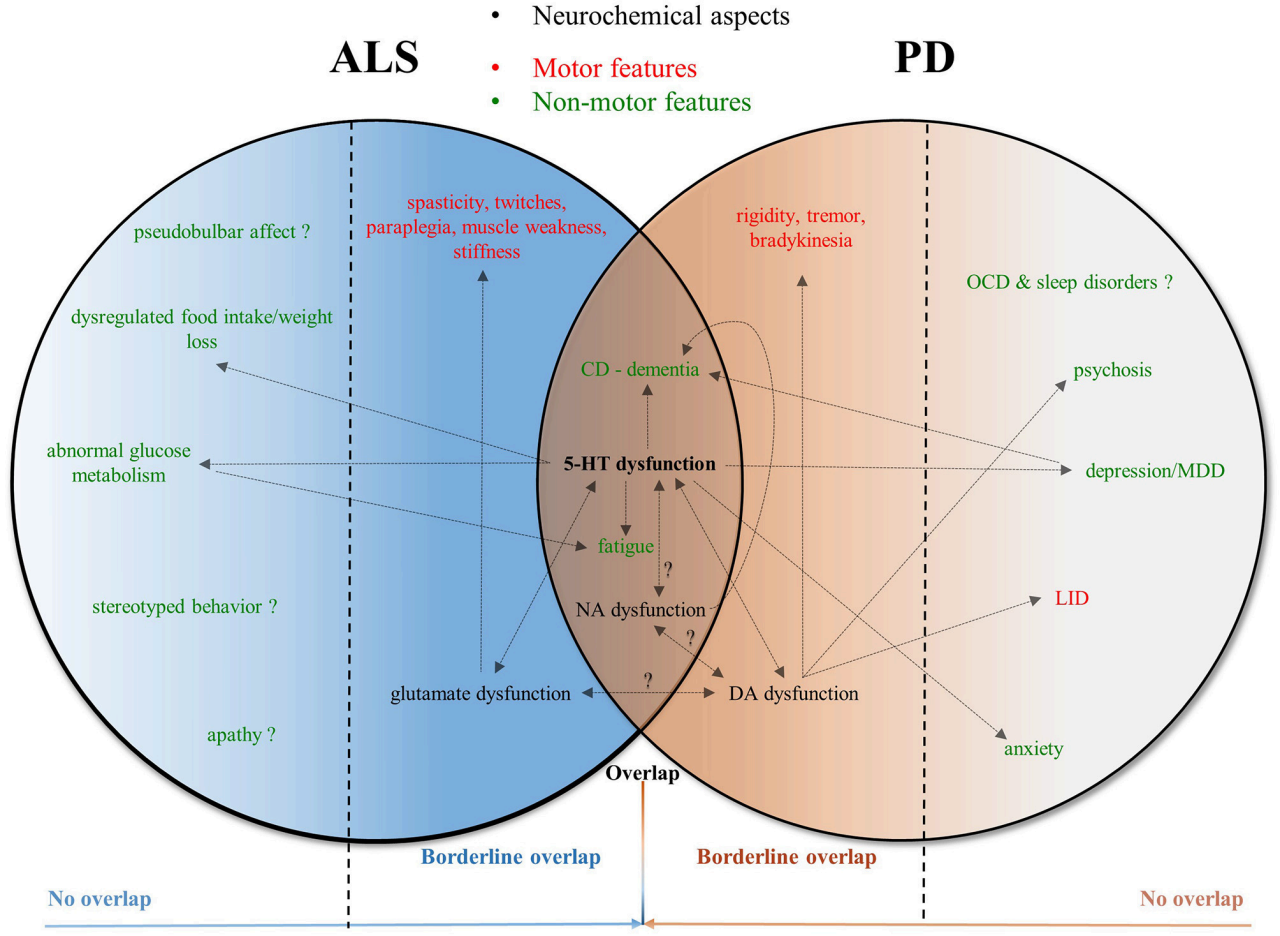
Venn diagram—visualization of the complex interplay of neurochemical and clinical keystones in ALS and PD. This figure depicts the complex interplay between neurochemical characteristics in ALS and PD related to their disease-specific clinical (i.e., motor and non-motor) outcome. Mutually influential and/or synergistic interactions are indicated with arrowheads at both ends. Question marks over the arrows refer to partially-proven or suggestive mechanisms of neurochemical features. The Venn diagram clearly shows that serotonergic dysfunction is the central overlap in the overall pathophysiology of ALS and PD, whereas the neurochemical causes of the clinical non-motor (i.e., behavioral) disturbances, particularly in ALS, necessitate further scrutiny. Similarly, the reciprocal interaction between the noradrenergic and serotonergic/dopaminergic disturbances in ALS and PD remains to be elucidated, even though in PD, NA dysfunction has been previously linked with CD and dementia progression. 5-HT, serotonin (5-hydroxytryptamine); ALS, amyotrophic lateral sclerosis; CD, cognitive deterioration; DA, dopamine; LID, levodopa-induced dyskinesia; MDD, major depressive disorder; NA, noradrenaline; OCD, obsessive-compulsive disorder; PD, Parkinson's disease.

The neurochemical similarity between ALS and PD, is that in both conditions, the supervising but damaged serotonergic system has lost pre- and postsynaptic regulatory functions on neighboring systems, leading to loss of inhibitory control of glutamate release and loss of facilitatory effects of glutamatergic motor neuron excitation in ALS, whereas in PD, this results in alterations of the complex serotonergic modulation of mesencephalic dopaminergic systems. Maybe unexpectedly, this largely and selectively affects upper and lower motor neurons in ALS brain and spinal cord, causing neuromuscular disease, while in PD, the effects rather remain central, i.e., at the level of the SNc/r, BG nuclei and (extra)striatal regions. Serotonergic alterations in ALS brain and RN have been found before (Turner et al., 2005), but the overall outcome of the serotonergic shortage on the corticocerebral level remains to be elucidated. In addition, autonomic and olfactory dysfunction in PD have been ascribed to peripheral noradrenergic alterations, potentially resulting from LC lesioning, and, likely, far preceding the motor deficits (Vermeiren and De Deyn, 2017). In contrast, there is a fairly dissimilar clinical outcome for both neurodegenerative diseases, with more 5-HT-associated non-motor disturbances in PD vs. a very typical motor—but less non-motor—region-dependent degenerative pattern in ALS, causing the well-characterized limb- or bulbar-onset phenotype (Figure 2).

Another very important peculiarity of 5-HT, which underscores its disease-influencing potential in ALS and PD, is its neuroprotective role through controlling energy homeostasis via still incompletely characterized mechanisms (Tecott, 2007). As such, new preclinical studies are emerging, which have already shown that 5-HT_1A/2B_ receptor stimulation on astrocytes and microglia promotes proliferation and upregulation of antioxidative molecules, slowing down or even reversing the disease process in ALS (El Oussini et al., 2016), and protecting dopaminergic neurons in PD (Miyazaki et al., 2013).

## Why it matters?

So far, there is total absence of easily-accessible biological markers in CSF or blood for ALS or PD, rendering the diagnosis of both disease entities sometimes fairly complex, laborious and challenging. Accordingly, the differential diagnosis among similar syndromes, including progressive supranuclear palsy, multiple system atrophy or corticobasal degeneration, may be quite difficult. Future studies should, therefore, focus on the serotonergic dysfunction in ALS and PD, and reveal if serotonergic markers alone or in combination with other biological factors, such as the LDL/HDL ratio, plasma ApoE, or various neuroinflammatory compounds (Dupuis et al., 2010), could be useful for routine diagnostic work-up of patients in clinics.

Additionally, serotonergic approaches in ALS and PD may alleviate disease burden on both the motor and non-motor level, and may hold great potential to influence the disease course, even though clinical trials with 5-HT modulating agents are currently scarce. Hypothetically, other neurodegenerative disorders, such as AD, dementia with Lewy bodies or PD plus syndromes, could—at least in part—share a fundamentally-alike monoaminergic pathophysiology, promoted by very early protein depositions in strategic brainstem nuclei. One might, therefore, wonder whether the universal quest for efficient symptomatic and disease-modifying therapies might, perhaps, be narrowed down to a monoaminergic-based derivative.

## Author contributions

All authors listed have made a substantial, direct, and intellectual contribution to the work, and approved it for publication.

### Conflict of interest statement

The authors declare that the research was conducted in the absence of any commercial or financial relationships that could be construed as a potential conflict of interest.
